# 837. Implementation of a Quick Reference Guide to Improve Wound Care Workflow

**DOI:** 10.1093/jbcr/irag033.276

**Published:** 2026-04-06

**Authors:** Jenna Rogers, Lisa C Smith, Yvette Jenifer, Emily H Werthman, Carrie A Cox, Cristian Cazac, Julie A Caffrey

**Affiliations:** Johns Hopkins Bayview Medical Center, Baltimore, MD; Johns Hopkins Bayview Medical Center, Forest Hill, MD; John’s Hopkins Medicine, Elkridge, MD; Johns Hopkins Burn Center, Baltimore, MD; Johns Hopkins Burn Center, Baltimore, MD; Johns Hopkins University School of Medicine, Baltimore, MD; Johns Hopkins University School of Medicine, Baltimore, MD

## Abstract

**Introduction:**

Burn care providers utilize keen observation skills, aseptic techniques, and carefully selected dressings to manage wounds and minimize infection risk. However, staying current with best practices can be challenging, as burn care continues to evolve. This quality improvement project aims to provide an educational resource that enhances providers’ understanding of current topical medications and dressing options for burn injuries.

**Methods:**

In June 2025, burn unit staff completed a brief anonymous survey assessing their perceptions and attitudes toward the existing wound care resources used in both clinical practice and education. All staff then received education on the Burn and Wound Medication and Dressing Quick Reference Guide, an educational tool developed to support wound care instruction and delivery. The guide features photos of each topical medication and dressing available at the burn center, along with detailed information on product descriptions, mechanisms of action, recommended uses, and manufacturer guidelines for application and frequency.

A follow-up survey was conducted in July and August 2025 to evaluate staff perspectives on the guide’s usefulness in daily workflow and wound care teaching. Laminated copies of the guide are readily accessible to all staff and are updated as product availability or recommendations change.

**Results:**

Prior to the intervention, 94% of staff relied on the wound care nurse or charge nurse as their main resource for wound care, with 51% describing their current resource as only somewhat helpful. When teaching others, 81% of staff depended on their work experience, and 56% rated their existing teaching resource as somewhat helpful. After introducing the quick reference guide, 72% of staff found it extremely helpful for wound care, and 67% felt it was extremely helpful when teaching others.

**Conclusions:**

Forty-five staff members completed pre- and post-surveys, highlighting a strong reliance on personal experience and peer guidance for wound care performance and teaching support. While this approach may be convenient for some, it can place excessive pressure on charge and wound care nurses. Additionally, depending solely on existing knowledge risks the continued use of outdated practices that may not align with current standards or advancements. Implementing a current, easily accessible wound care resource can help streamline workflow, support clinical decision-making, and enhance both the delivery and teaching of wound management.

**Applicability of Research to Practice:**

Up-to-date knowledge and evidence-based practices are essential for the comprehensive care of burn injuries. Utilizing reliable, current resources empowers staff to provide consistent, high-quality care and can lead to improved patient outcomes.

**Funding for the study:**

N/A.

